# A Propensity-Score Matched Comparison of Perioperative and Early Renal Functional Outcomes of Robotic versus Open Partial Nephrectomy

**DOI:** 10.1371/journal.pone.0094195

**Published:** 2014-04-07

**Authors:** Zhenjie Wu, Mingmin Li, Le Qu, Huamao Ye, Bing Liu, Qing Yang, Jing Sheng, Liang Xiao, Chen Lv, Bo Yang, Xu Gao, Xiaofeng Gao, Chuanliang Xu, Jianguo Hou, Yinghao Sun, Linhui Wang

**Affiliations:** 1 Department of Urology, Changhai Hospital, Second Military Medical University, Shanghai, P. R. China; 2 Department of Radiology, Changhai Hospital, Second Military Medical University, Shanghai, P. R. China; H. Lee Moffitt Cancer Center & Research Institute, United States of America

## Abstract

**Objectives:**

To compare the perioperative and early renal functional outcomes of RPN with OPN for kidney tumors.

**Materials and Methods:**

A total of 209 RPN or OPN patients with availability of preoperative cross-sectional imaging since 2009 at our center were included. To adjust for potential baseline confounders propensity-score matching was performed, which resulted in 94 OPNs matched to 51 RPNs. Perioperative and early renal functional outcomes were compared.

**Results:**

In propensity-score matched analysis, RPN procedures were well tolerated and resulted in significant decreases in postoperative analgesic time (24 vs. 48 hr, *p*<0.001) and visual analog pain scale (3 vs. 4, *p*<0.001). Besides, the RPN patients had a significantly shorter LOS (9 vs. 11 days, *p* = 0.008) and less EBL (100 vs. 200 ml, *p*<0.001), but median operative time was significantly longer (229 vs. 182 min, *p*<0.001). Ischemia time, transfusion rates, complication rates, percentage eGFR decline and CKD upstaging were equivalent after RPN versus OPN. In multivariable logistic regression analysis, RPN patients were less likely to have a prolonged LOS (odds ratio [OR]: 0.409; *p* = 0.016), while more likely to experience a longer operative time (OR: 4.296; *p* = 0.001). However, the statistical significance for the protective effect of RPN versus OPN in EBL was not confirmed by examining the risk of EBL≥400 ml (OR: 0.488; *p* = 0.212).

**Conclusions:**

When adjusted for potential selection biases, RPN offers comparable perioperative and early renal functional outcomes to those of OPN, with the added advantage of improved postoperative pain control and a shorter LOS.

## Introduction

Partial nephrectomy (PN) has become the established standard treatment for most localized small renal tumors in that it yields equivalent long-term oncological outcomes and better preservation of renal function compared to those of radical nephrectomy (RN) [1]–[3]. Moreover, a large population-based analysis by Tan et al revealed that cancer-specific survival rates following PN and RN in patients with early-stage kidney cancer are comparable and PN is associated with an improved overall survival rate [4].

Of currently available PN techniques, open surgery remains a standard of care for PN [1]. While in areas other than PN, laparoscopic surgery appears to have definite advantages in reduced surgical invasiveness and postoperative recovery which would not be offset by worse function outcomes of the organ operated or increased perioperative complication profiles relative to open surgery [5]–[7]. Despite its mini-invasiveness and excellent results in experienced hands, laparoscopic PN (LPN) is reported to be associated with a prolonged warm ischemia time and a higher complication rate than with OPN [8], [9]. It is the increased technical difficulty as well as the steep learning curve that limits the diffusion of LPN. In contrast, robotic PN (RPN) appears to be a more reproducible approach with improved dexterity, magnified three-dimensional visualization and better ergonomics, which may bridge the gap between the LPN and OPN. A recently published meta-analysis on RPN vs. LPN showed a significantly decreased warm ischemia time with RPN and comparable outcomes in terms of operative time, estimated blood loss, length of stay, complication or positive margins. As RPN is increasingly gaining popularity, a rigorous comparison against the gold standard of OPN is desperately needed. Even though several observational studies comparing RPN and OPN have been recently reported, most are significantly suffering from the confounding of salient baseline covariates with conflicting results [10]–[13]. Therefore, we aimed to evaluate the effects of RPN on perioperative and early renal functional outcomes relative to OPN based on a propensity-score matched cohort.

## Materials and Methods

### Patient and Measurement

Following the approval of our Institutional Review Board (IRB) of Changhai hospital (Second Military Medical University, SMMU), the electronic medical record system and the radiological database (Picture Archiving and Communication Systems, PACS) were queried to identify all patients with preoperative cross-sectional imaging (enhanced computerized tomography or magnetic resonance imaging) who underwent RPN or OPN from 2009 to 2013 at a tertiary reference center. All radiological images were reviewed and evaluated electronically by a senior radiologist (MML) and an experienced urologist (ZJW) who were blinded to the surgical approaches and outcomes. Tumor size was recorded as the largest diameter on the axial plane. For each renal tumor a diameter-axial-polar (DAP) score ranging from 3 to 9 points was assigned according to the reported methodology [14]. Hilar lesions were determined according to the definition in the R.E.N.A.L. nephrometry system [15].

Surgical approach and technique, eg. OPN or RPN, were chosen according to the primary surgeon's judgment instead of randomization. All procedures were performed by surgeons with advanced training in open and minimally invasive surgery. RPN operations were conducted with the da Vinci Si Surgical System (Intuitive Surgical, Inc., Sunnyvale, CA, USA) by one of four surgeons with substantial experience in partial nephrectomy. Patients were placed in flank position. A standard three-arm approach with one or two trocars for the assistant, and an optional robotic port as needed was used. After the Gerota's fascia was opened, the renal vessels were dissected and the tumor was identified with the assistance of intraoperative ultrasonography as needed. Most of the hilar controlled RPN procedures were performed with renal artery-only clamping. Tumor resection was performed with a tumor-free parenchymal margin of 0.5–1 cm in thickness. Standard OPN procedures were done, with flank incisions between the 10^th^ or 11^th^ interspace. The renal pedicle was usually controlled en bloc with a vascular clamp. Cold ischemia with ice slush was used. In all open and robotic cases, opened calices and bleeding sites were repaired carefully. The parenchymal defect was closed using a combination of sliding-clip (Hem-o-lok) renorrhaphy and a running suture. Additional absorbable hemostatic agents were used when necessary. When necessary, patients received continuous intravenous analgesics for a maximum of 48 hours after surgery. For the pain assessment, the visual analog scale (VAS) was used, which ranges from 0 (no pain) to 10 (excruciating pain).The pain scale was self-administered on first three postoperative days by the nurse during the morning round. The highest pain score was included for analysis.

Perioperative data analyzed included patient age, gender, body mass index (BMI), American Society of Anesthesiologists (ASA) classification, age-weighted Charlson Comorbidity Index (CCI) score, DAP nephrometry score as well as tumor size, operative duration, estimated blood loss (EBL), ischemia time, proportion of patients with intraoperative collecting system entry, hemostatic agent use, perioperative transfusions and complications, conversions to radical nephrectomy, postoperative analgesic time, pain scale, length of hospital stay (LOS), surgical margin status and eGFR change. Positive surgical margin was defined as cancer cells at the level of inked parenchymal excision surface. The eGFR was calculated using the Modification of Diet in Renal Disease 2 equation [16]. For baseline eGFR the SCr value prior to surgery (generally within one week before surgery) was used. The last eGFR measurement was within 3 months after surgery. The follow-up SCr value for the last eGFR measurement was obtained by searching the lab testing system with the patient name, patient identity number or inpatient number, and otherwise the SCr most immediately preceding discharge was used. For each patient, chronic kidney disease (CKD) was defined according to the National Kidney Foundation Kidney Disease Outcome Quality Initiative classification [17]. The upstaging of CKD was considered as a change in one class of CKD or more. Percentage eGFR change was defined as (last eGFR-baseline eGFR)/baseline eGFR. Postoperative complications were graded according to the Clavien scale. The PACS database was queried for the oncological outcome analysis. Patients who had stopped follow-up at our institution were contacted by telephone to inquiry their latest imaging results. For those who did not respond, the most recent follow-up images within the PACS were used.

### Statistical Analysis

Prior to analysis, patient information was anonymized and de-identified. Categorical variables were shown as the frequency and percentage, and continuous variables were presented as the median and interquartile range (IQR). Frequency distributions between categorical variables were compared usingχ^2^ test or Fisher's exact test while continuous variables were compared using the nonparametric Mann-Whitney test. Due to inherent differences between patients who underwent RPN and OPN in terms of baseline characteristics, we performed a propensity score matched analysis to adjust for these differences. Exclusion criteria for propensity score analysis of the RPN versus OPN groups were as follows (numbers represent counts of patients): solitary kidney patients (2, 6), multiple ispilateral tumors (0, 8), history of partial nephrectomy in the contralateral kidney (0, 4), “zero ischemia” (off-clamp or segmental branches clamped) patients (1, 1), missing ischemia time (0, 6), and missing postoperative eGFR measurement (0, 2).

The probability to undergo a RPN procedure in the current study was estimated. The propensity score was generated by way of a multivariable logistic model considering the following variables: patient demographics (age, gender, BMI); ASA score; CCI; hilar tumor; DAP sum score; and preoperative eGFR level. Based on the resulting propensity score, one case was matched to one or multiple controls using a caliper of 0.01. Subsequently, covariate balance and surgical outcomes between the matched groups were examined. Finally, backward stepwise logistic multivariable regression analysis were conducted for prediction of several outcomes, namely, the odds of operative time of 4 hr or more, EBL of 400 ml or greater, ischemia time of 20 min or longer, a prolonged LOS (≥10 d), any postoperative complication during hospital stay, postoperative eGFR decrease ≥10% and any CKD upstaging. All statistical analyses were performed using the IBM SPSS Statistics v.20. The null hypothesis was rejected for all analyses at *p*<0.05, and all *p* values were 2-tailed.

## Results

Overall, 209 patients were included, of which 123 (58.9%) and 86 (41.1%) patients were treated by OPN or RPN, respectively. There was one intraoperative conversion of RPN to OPN due to hemorrhage and one conversion to radical nephrectomy in the OPN group for oncological reason (report of malignance in the frozen section analysis). There was no death (Clavien 5) from surgical complication in either group. No patients had positive surgical margin. The median percentage decrease in eGFR at a median of 3 months was 6% (IQR: -20-4) and no patients required dialysis. The median oncological follow-up was 12 months (IQR: 6–24). One patient (0.8%) of OPN with clear cell renal cell carcinoma (Fuhrman III) had local recurrence at three years after surgery and underwent radical nephrectomy. There were no metastatic diseases developed in the study cohort.

Prior to matching, more patients in the OPN group had a hilar tumor (16.3% vs 7%, *p* = 0.045) and a higher DAP nephrometry score (7 vs 6.5, *p* = 0.037) compared with that of the RPN group. No differences according to age, gender, BMI, ASA, age-adjusted CCI and preoperative eGFR were detected between the two groups. Propensity-score matching was subsequently performed, which resulted in a cohort including 51 RPN and 94 OPN patients. As expected, differences in patient characteristics between the two groups were all non-significant, indicating a high degree of similarity in the distribution of potential confounders (Table 1).

**Table 1:** Distribution of potential confounders used in the propensity-score model in patients before and after matching.

Median (Q_1_–Q_3_) or n(%)	Entire sample				Propensity-score matched groups			
	Total	RPN	OPN	*p* value	Total	RPN	OPN	*p* value
No. of pts	209	86	123	**-**	145	51	94	**-**
Age, yr	50	52	49	0.141	52	53	52	0.750
	(42–59)	(43–60)	(41–59)		(46–60)	(45–59)	(46–60)	
Gender				0.155				0.744
Male	129(61.7)	58(67.4)	71(57.7)		97(66.9)	35(68.6)	62(66)	
Female	80(38.3)	28(32.6)	52(42.3)		48(33.1)	16(31.4)	32(34)	
BMI, kg/m^2^	24.6 (22.3–26.2)	24.7 (22.9–26.2)	24.2 (21.3–26.3)	0.341	24.9 (22.8–26.4)	24.8 (22.8–26.2)	25.1 (22.9–26.6)	0.762
ASA				0.705				1.000
1–2	196(93.8)	80(93)	116(94.3)		136(93.8)	48(94.1)	88(93.6)	
3–4	13(6.2)	6(7)	7(5.7)		9(6.2)	3(5.9)	6(6.4)	
Age-adjusted CCI				0.830				0.305
0–1	181(86.6)	75(87.2)	106(86.2)		125(86.2)	46(90.2)	79(84)	
≥2	28(13.4)	11(12.8)	17(13.8)		20(13.8)	5(9.8)	15(16)	
Hilar tumor	26(12.4)	6(7)	20 (16.3)	**0.045**	10(6.9)	3(5.9)	7(7.4)	1.000
DAP sum score	7 (6–7)	6.5(5–7)	7(6–8)	**0.037**	7 (5–7)	6 (5–7)	7 (5–7)	0.370
Preoperative eGFR (ml/min per 1.73 m^2^)	98 (86.6–110.3)	96.5 (86.1–108.7)	98.4 (87.5–112.1)	0.425	97 (86.4–106.5)	95.5 (88.3–104.5)	97.5 (85.0–109.1)	0.828

IQR  =  interquartile range; RPN  =  robotic partial nephrectomy; OPN  =  open partial nephrectomy; ASA  =  American Society of Anesthesiologists; CCI  =  Charlson Comorbidity Index; DAP  =  diameter-axial-polar.

Within the propensity-score matched cohort (Table 2), RPN procedures were well tolerated and resulted in significant decreases in postoperative analgesic time (24 vs. 48, *p*<0.001) and visual analog pain scale (3 vs. 4, *p*<0.001). Besides, RPN patients had less EBL and a shorter LOS, whereas the OR time was significantly longer (229 vs. 182, *p*<0.001) and more patients encountering delayed bleeding (7.8% vs. 5.5%, *p* = 0.030) than that of OPN patients. The differences between the two groups were not statistically significant with regard to ischemia time, proportion of patients with intraoperative collecting system entry, hemostatic agent use, transfusion rates, overall complication rate, the rate of urine leak, postoperative eGFR decrease in percentage or CKD upstaging. The propensity-score adjusted multivariable logistic regression analysis showed that relative to OPN patients, RPN patients were less likely to have a prolonged LOS (≥10 d) following surgery (odds ratio [OR]: 0.409; 95% confidence interval [CI]: 0.198–0.845; *p* = 0.016), while more likely to experience a longer operative time (≥4 h) (OR: 4.296; 95% CI: 1.870–9.871; *p* = 0.001). However, the statistical significance for the protective effect of RPN versus OPN in EBL was not confirmed by examining the risk of EBL≥400 ml (OR: 0.488; 95% CI: 0.158–1.506; *p* = 0.212) and the higher risk of delayed bleeding for RPN did not reach statistical significance(OR: 1.834; 95% CI: 0.440–7.643; *p* = 0.405). Finally, no statistically significant differences were detected regarding ischemia time of 20 min or longer (OR: 0.954; *p* = 0.905), postoperative complication rate (OR: 1.654; *p* = 0.247), eGFR decline≥10% (OR: 1.002; *p* = 0.996), or CKD upstaging (OR: 0.977; *p* = 0.954) (Table 3).

**Table 2:** Propensity-score adjusted comparison of surgical outcomes for RPN and OPN.

Median (Q_1_–Q_3_) or n(%)	RPN (n = 51)	OPN (n = 94)	*p* value
OR time, min	229 (203–268)	182(161–223)	**<0.001**
EBL, ml	100 (100–200)	200(113–300)	**<0.001**
Ischemia time, min	21 (15–27)	20 (17–27)	0.899
LOS, d	9 (8–12)	11 (9–13)	**0.008**
Blood transfusion	3 (5.9)	4 (4.3)	0.697
Collecting system entry	14(27.5)	27(29.7)	0.871
Hemostatic agent use*	42(82.4)	73(77.7)	0.505
Intraoperative complication	1 (2)	1 (1.1)	1.000
Conversion to radical nephrectomy	0 (0)	1 (1.1)	1.000
VAPS (0–10)	3 (3–4)	4 (4–6)	**<0.001**
Postoperative Analgesic time, hr	24 (19–24)	48 (24–48)	**<0.001**
Postoperative complication			
Overall	13 (25.5)	17 (18.1)	0.293
Clavien 1–2	12 (23.5)	16 (17)	0.343
Clavien 3–5	1 (2)	1 (1.1)	1.000
Delayed bleeding#	4(7.8)	5(5.5)89	**0.030**
Urine leakξ	1(2)	2(2.1)	0.613
Positive surgical margin	0 (0)	0 (0)	-
Last eGFR, ml/min per 1.73 m^2^	92.4 (76.7–102.5)	88.5 (74.9–100.5)	0.561
Percentage change in eGFR	−6 (−18–3)	−8 (−21–2)	0.744
Preoperative CKD 1–2	50 (98)	91 (96.8)	0.666
Postoperative CKD 1–2	46 (90.2)	87 (92.6)	0.754
CKD upstaging	15 (29.4)	29 (30.9)	0.857

IQR  =  interquartile range; RPN  =  robotic partial nephrectomy; OPN  =  open partial nephrectomy; OR  =  operative room; EBL  =  estimated blood loss; LOS  =  length of stay; VAPS  =  visual analog pain scale; CKD  =  chronic kidney disease.

*SURGICEL.

# defined as a decreased level of Hb requiring blood transfusion or surgical/endoscopic/radiologic intervention 24 hr after surgery or later.

ξ defined as extra-renal urine extravasation that required prolonged maintenance of a drain, re-insertion of a drain, insertion of a ureteral stent or other surgical intervention. All leaks were verified by drain fluid chemical analysis. Cases of urinary leak in both RPN and OPN groups were managed expectantly.

**Table 3:** Propensity-score adjusted multivariable stepwise logistic regression analysis for surgical outcomes of RPN versus OPN.

Dependent variable	RPN vs OPN: odds ratio (95% CI)*	*p* value
OR time ≥4 hr	4.296 (1.870–9.871)	**0.001**
EBL ≥400 ml	0.488 (0.158–1.506)	0.212
Ischemia time ≥20 min	0.954 (0.439–2.074)	0.905
LOS ≥10 d	0.409 (0.198–0.845)	**0.016**
Postoperative complication	1.654 (0.706–3.876)	0.247
Delayed bleeding	1.834(0.440–7.643)	0.405
eGFR decrease ≥10%	1.002 (0.491–2.045)	0.996
CKD upstaging	0.977 (0.453–2.110)	0.954

RPN  =  robotic partial nephrectomy; OPN  =  open partial nephrectomy; OR  =  operative room; EBL  =  estimated blood loss; LOS  =  length of stay; CKD  =  chronic kidney disease.

*Models adjusted for age, gender, baseline Charlson comorbidity index, BMI, ASA, and DAP score.

## Discussion

The present study yielded some important findings. In comparison with OPN, RPN provided comparable perioperative and functional outcomes with the added benefits of better postoperative pain control and a shorter postoperative hospital length of stay. Specifically, RPN patients are 40.9% less likely to experience a prolonged LOS and have a marginal advantage in EBL without compromising surgical success relative to OPN, albeit with a four-fold risk of operative time over 4 hours.

Four other reported studies evaluated surgical outcomes after RPN versus OPN [10]–[13]. In those series, the treatment-selection bias was not balanced, although two of them announced prospective design and data collection. For example, percentage of imperative indication for PN [10], [13], CCI [10], BMI [11], tumor size [10], or tumor anatomic complexity [12] significantly differed between the two groups. The crude comparison might reflect a “real world” scenario that more patients with complex renal tumors underwent open surgery, as showed in our unmatched series. However, the heterogeneity in baseline characteristics would mix the impact of treatment on outcomes. Many studies reported that differences in tumor complexity, BMI, or CCI may account for the observed differences in outcomes [18]–[21], which reinforces the necessity that statistical methods must be used to adjust for systematic differences.

The current data with propensity-score adjustment to achieve a minimum inherent selection bias according to treatment type may help to address the controversy on ischemia time with RPN versus OPN. Lee et al reported a longer ischemia time in the RPN group (23 vs. 19 min, *p*<0.001) [11]. In accordance with our results, the study by Simhan et al demonstrated comparable ischemia time following RPN and OPN [13], which was also confirmed in two prospective non-randomized comparative studies [10], [12]. In this regard, surgeons' experience in RPN also contributes to the reduction in intraoperative ischemia time. In several recently reported series of RPN for complex renal tumors (a higher nephrometry score, multifocal, or completely endophytic, etc.), the ischemia time can still be controlled at about 20 min [22]–[24]. Regarding the postoperative renal functional outcome, a large tertiary-care center series comparing RPN and OPN revealed that there was no significant difference in eGFR change at a mean follow-up of 21.3 months. In that study, however, more patients with solitary kidney were treated with OPN (12.1 vs 0%, *p*<0.001), which can intrinsically influence the estimation of eGFR in the OPN group. Similarly, Lee et al and Masson-Lecomte et al proved the equipoise between RPN and OPN [11], [12]. In our study, the comparable early renal functional outcome was confirmed and no increased risk of CKD upstaging was associated with RPN. Recently, a novel method for renal hypothermia during RPN that recapitulates the open approach has been under further evaluation, which would contribute to a better functional preservation [25].

More recently, Ficarra et al reported a multicenter matched-pair analysis comparing robotic versus open PN, in which 200 RPNs and 200 OPNs were examined [26]. In that series, EBL and LOS were more favorable after RPN than OPN, and no differences were recorded regarding intraoperative complications (1 vs. 3, *p* = 0.31), blood transfusions (21 vs. 20, *p* = 0.78), high grade (Clavien 3–4) postoperative complications (9 vs. 9, *p* = 1.000), and absolute eGFR decline at 3 months after surgery (16.6 vs. 16.4 ml/min, *p* = 0.28). These data are in perfect agreement with our findings, but the protective effect of RPN in EBL (100 vs. 150 ml, *p*<0.001 in their cohort; 100 vs. 200 ml, *p*<0.001 in our series) is marginal. What's more, in the procedure of RPN the amount of blood loss could be underestimated for blood loss might not be fully recognized due to gravity effects on the blood into more dependent abdominal compartments that go unrecognized and “unsuctioned” from the body cavity. This effect may be of little clinical significance for it is not predictive of EBL≥400 ml in multivariable analysis. In this regard, it may be better to assess the change in perioperative hemoglobin or hematocrit. However, there could be differences in hydration status in the perioperative setting that might affect accurate measurement.

It is equally interesting to note that there was no significant differences between the two approaches in operative time but a significantly longer warm ischemia time with RPN than with OPN in Ficarra et al's study. Actually, in their series the OPN patients were collected from 19 different Italian centers while most RPN patients were captured from databases abroad. The resulted heterogeneity in surgical experience and technique could lead to biased estimates of treatment effects. The majority of our series were performed by the consultant surgeon of the kidney tumor specialized group at our institution, although RPN is considered to have a short learning curve in the hands of a surgeon with extensive minimally invasive surgery experience [27]. The longer operative room time with RPN in our series might be explained by the inclusion of the time for preparation and docking of the robot [12].

The current study highlights the non-inferiority of RPN to OPN with regard to intraoperative ischemia time, postoperative complications and early functional outcomes and the additional benefits of a shorter LOS as well as less EBL using propensity-score matched comparison and prediction analysis. In spite of its strengths, our report has limitations inherent in its retrospective nature. Some unmeasured data which were not retrospectively retrievable may be of paramount importance, especially with regard to time off take-in, drainage, time required for patients return to their occupations, etc., which may favor one approach over another. There may also have been some unobserved bias amongst the groups such as surgeons' background or intraoperative technique that we were not able to adjust for. The differences in the ischemia type between the two groups, and utilization of the Modification in Diet Renal Disease 2 equation for eGFR which is affected by diet would cause potential bias in the evaluation of renal functional impairment. Besides, missing data fields which led to further exclusion of cases could decrease the power of our study. Also, the few events of major postoperative complications may eliminate the potential differences between the two groups. Finally, the oncological outcomes were not compared directly because the follow-up duration between RPN and OPN were significantly different.

## Conclusions

When adjusted for potential selection biases, RPN offers comparable perioperative and early renal functional outcomes to those of OPN, with the added advantage of improved postoperative pain control and a shorter length of hospital stay. However, these results should be interpreted with caution, given that statistical adjustment is not a substitute for an awaited randomized trial. A long-term follow-up is needed to confirm the oncological safety and efficacy of RPN.
